# An unusual case of infected *uterus masculinus* in a dog

**DOI:** 10.1186/s12917-020-02396-2

**Published:** 2020-06-15

**Authors:** Michal Kyllar, Petr Čížek

**Affiliations:** 1 Companion Care Veterinary Surgery, East Kent Retail Park, Westwood Road, Broadstairs, Kent, CT10 2RQ UK; 2 Department of Anatomy, Histology and Embryology, Faculty of Veterinary Medicine, University of Veterinary and Pharmaceutical Sciences, Brno, Czech Republic

**Keywords:** Paraprostatic cyst, Prostate, Prostatitis, Sertoli cell tumour,

## Abstract

**Background:**

Paraprostatic cysts are large structures that develop between the prostate gland and urinary bladder, usually in older, intact dogs. Their incidence is reported to be 1.1–5.3% in dogs with prostatic disease. The aetiology of paraprostatic cysts is not fully understood, but they are believed to develop from the *uterus masculinus*. Whereas the *uterus masculinus* has been reported to communicate with the urethra in men and horses, no communication between the *uterus masculinus* and urethra has been identified in dogs.

**Case presentation:**

An entire male dog was presented with a bloody discharge from its penis and tenesmus of 5 days’ duration. A diagnosis of cystic *uterus masculinus* was made on the basis of the findings of abdominal ultrasonography and histopathology of tissues obtained during an exploratory laparotomy. In addition, a Sertoli cell tumour affecting both testes was diagnosed following scrotal castration. The cystic *uterus masculinus* was completely resected, after which the tenesmus and bloody discharge resolved. Thus, cystic *uterus masculinus* should be considered as a differential diagnosis for a paraprostatic cyst when such a lesion develops as part of the feminising effect of a Sertoli cell tumour.

**Conclusions:**

Cystic *uterus masculinus* should be considered as a differential diagnosis for tenesmus and penile discharge, and for structures resembling paraprostatic cysts. This case report confirms that a *uterus masculinus* can communicate with the urethra in dogs, as in other species, and demonstrates endocrine responsiveness, manifesting as epithelial and glandular metaplasia and mucus production, with the potential for subsequent infection.

## Background

Paraprostatic cysts are large structures that develop between the prostate gland and urinary bladder [1]. They usually arise from the craniodorsal aspect of the prostate gland and extend cranially. These cysts have very little association with the prostate gland itself and only rarely communicate with the urethra via the prostate [2–4]. Paraprostatic cysts are usually diagnosed in older dogs [4, 5]; their incidence is reported to range from 1.1 to 5.3% in dogs with prostatic disease, and all the reported cases have been intact males [6–8]. The aetiology of paraprostatic cysts is not fully understood, but are considered to be of the *uterus masculinus* origin, which is a remnant of the Müllerian tube [4, 9, 10]. As the hollow cavity develops, it remains connected with the prostate gland. Affected animals are frequently without clinical signs. Clinical symptoms usually develop with an enlargement of its cavity but as the cyst increases in size, and may include tenesmus, abdominal enlargement associated with pain and in progressed cases a stranguria [1, 3, 5, 6].

The treatment of paraprostatic cyst in dogs may involve drainage, resection or marsupialization [6, 10–12].

## Case presentation

A 12-year-old entire male Basset Hound presented with a 1 week history of tenesmus, bloody penile discharge and anorexia, and polyuria and polydipsia of 2 days’ duration. General examination revealed mild dehydration and a high rectal temperature (39.5 °C). Digital rectal examination and caudal abdominal palpation revealed an enlarged, symmetrical and painful mass that extended from the cranial hypogastrium to the pelvic cavity, just cranial to the prostate. The prostate was smooth, non-painful and of a normal size. Scrotal palpation revealed diffusely swollen scrotal tissue and vaginal tunics, and small, solid, non-painful testes. There was a mucopurulent discharge from the external orifice of the urethra.

Haematology revealed mild non-regenerative anaemia, leucocytosis and neutrophilia with left shift. A serum biochemistry panel yielded results consistent with dehydration: slight increases in alanine aminotransferase (112 U/L; reference range: 20–98 U/L), alkaline phosphatase (125 U/L; reference range: 17–111 U/L) and hyperglobulinaemia (78 g/L; reference range: 24–40 g/L) were noted. Subsequent endocrine evaluation revealed a low serum testosterone concentration (0.9 ng/ml; reference range: 1.5–8.5 ng/ml; radioimmunoassay) and high concentrations of oestradiol (32 pg/ml; reference range: < 15 pg/ml; radioimmunoassay) and progesterone (3.1 ng/ml; reference range for neutered females: < 1.0 ng/ml; radioimmunoassay).

**Fig. 1:**
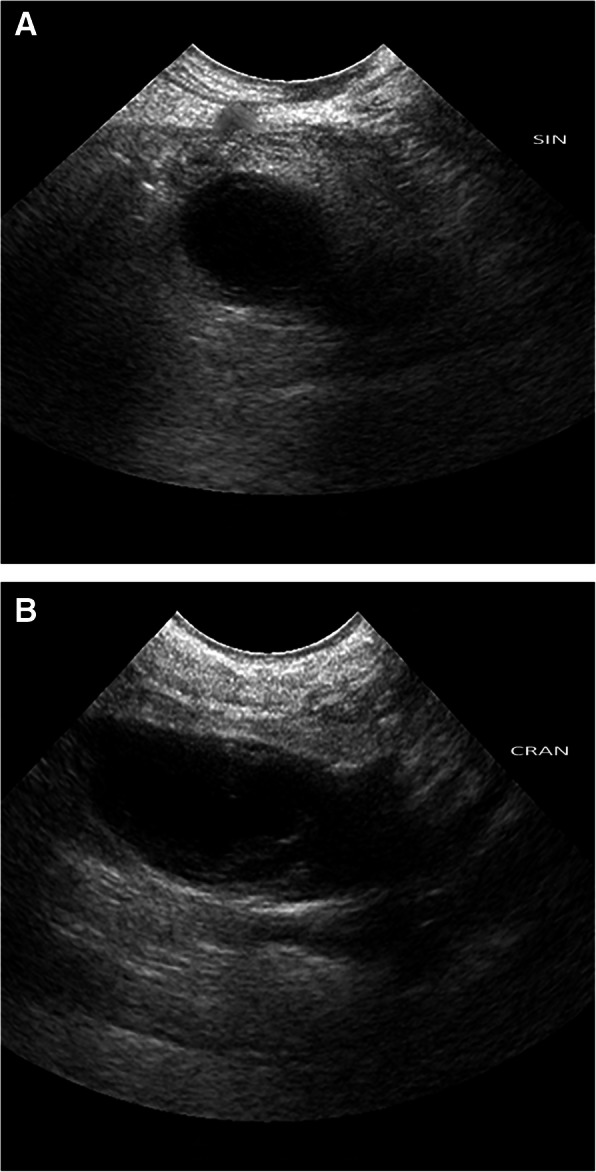
Ultrasonography of the *uterus masculinus* Transverse (**a**) and sagittal (**b**) ultrasonographic images of the *uterus masculinus*, showing the hypoechoic fluid within

**Fig. 2:**
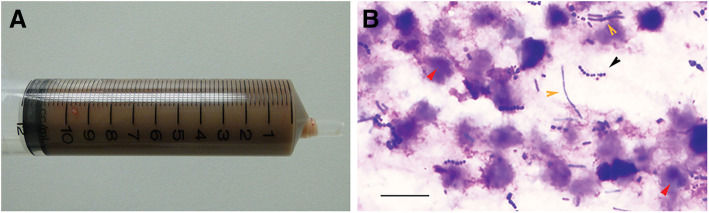
Cystocentesis of the uterine contents **a** Macroscopic appearance of the uterine contents. **b** Cytological examination revealed the cellular component to consist predominantly of degenerated neutrophils (red arrowhead), cocci (black arrowhead) and rod-shaped bacteria (yellow arrowhead). Bar: 50 μm

Abdominal ultrasonography revealed a hypoechoic hollow lesion that was 10 cm in length and up to 4 cm wide, which was adjacent to the prostate and extended cranially, beyond the level of the apex of the urinary bladder (Fig. 1a–b). The prostate was normoechoic and had a diameter of 2.5 cm. The urinary bladder was moderately full and had a wall of normal thickness, without irregularities. Cystocentesis of the urinary bladder was performed, and the fluid obtained was submitted for biochemical and cytological evaluation, as well as culture and sensitivity testing. The urine was clear, and had a pH of 7.0 and a specific gravity of 1.020. The urine cytology was normal and the bacterial culture was negative. Cystocentesis of the cystic structure revealed that it was filled with a dark mucopurulent liquid (Fig. 2a). Cytological examination of this revealed abundant mature and degenerate neutrophils, a small number of large polygonal epithelial cells and a low N:C ratio (Fig. 2b). Clusters of rods and cocci were also found. Subsequent culture of the fluid showed the presence of *Escherichia coli*.

**Fig. 3:**
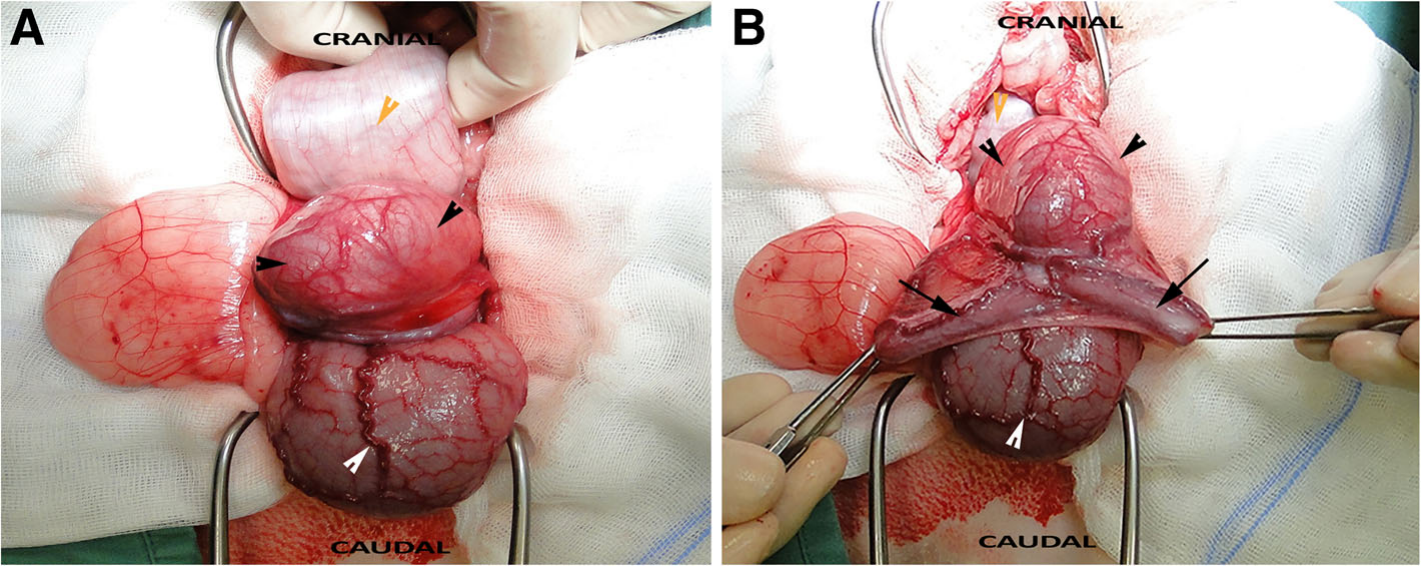
Surgical exploration and removal of the *uterus masculinus* Fluid-filled *uterus masculinus*. **a** The body (black arrowhead) was located between the urinary bladder (white arrowhead) and descending colon (yellow arrowhead). **b** The uterine horns (black arrows), which arose from the body of the *uterus masculinus*

Exploratory laparotomy revealed the presence of a single, large paraprostatic cyst that was located dorsal to the neck of the urinary bladder, arose from the prostate, extended cranially into the abdominal cavity, and had two horn-like blind-ended tubes that were suspended from the genital fold (Fig. 3a–b). The *ducti deferentia* lay lateral to the cyst. The cyst was ligated and fully resected, and the abdominal cavity was closed in routine fashion. The testes were removed via a scrotal approach and then scrotal ablation was performed. Both testes were firm and small (2 cm in diameter and 3.5 cm in length). A 10 day course of oral amoxicillin/clavulanic acid (Nisamox, Norbrook; Newry, United Kingdom; 12.5 mg/kg twice daily) was then administered and post-operative analgesia was provided using meloxicam (Metacam, Boehringer Ingelheim; Germany; 0.1 mg/kg once daily for 7 days).

The tenesmus and penile discharge resolved immediately following the surgery and examinations conducted 10, 30, and 60 days later were unremarkable. Ultrasonography performed 60 days after surgery revealed normal hypogastric and pelvic anatomy.

**Fig. 4:**
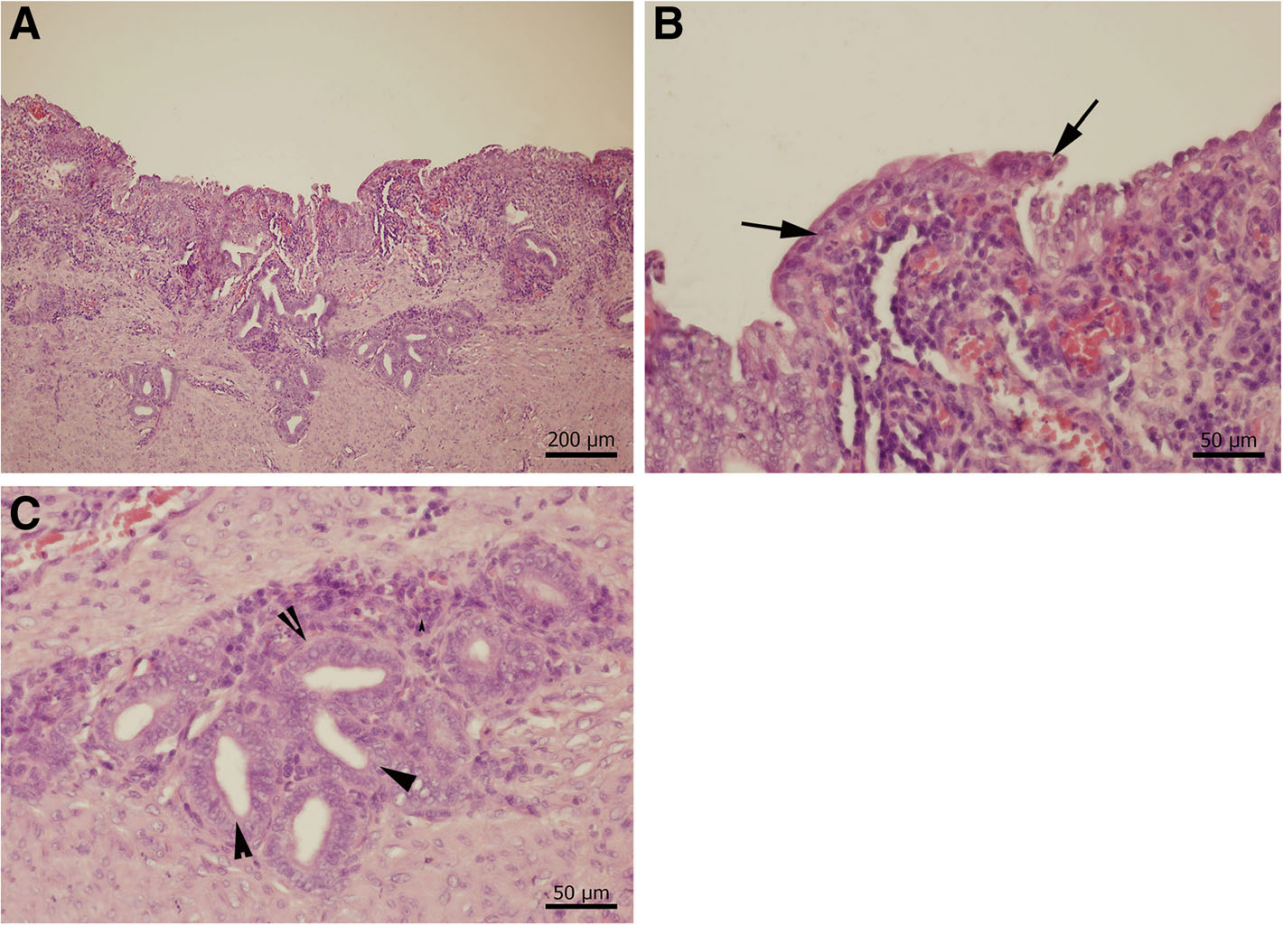
Histopathological assessment of the uterus masculinus **a**, Cross section of the wall of the uterus masculinus with characteristic appearance of a tubular structure with simple columnar epithelium, glandular structures, smooth muscle layer and inflammatory infiltration; **b**, Detail of the simple columnar epithelium (black arrows) (bar – 50 μm); **c**, Detail of the glandular structures - black arrowheads (bar – 50 μm)

Histopathology of the paraprostatic cyst showed that it was a tubular structure, lined by simple columnar epithelium that contained glands, and therefore resembled endometrium. It also had a smooth muscle layer, but no evidence of neoplasia. Mild lymphocytic, neutrophilic, plasmacytic and eosinophilic inflammatory cell infiltration was also present. A histological diagnosis of true *uterus masculinus* was made (Fig. 4a–c). Histopathological examination of sections prepared from the testes revealed the presence of neoplastic changes in the seminiferous tubules. The mass was composed of neoplastic intratubular Sertoli cells and a significant scirrhous reaction. The cells showed mild anisocytosis and anisokaryosis, were fusiform in shape and contained a moderate amount of eosinophilic cytoplasm, with variably distinct cellular borders. Their nuclei were oval and euchromatic, and contained stippled chromatin and a single basophilic nucleolus. Three mitotic figures were present. Therefore, a histological diagnosis of bilateral Sertoli tumour was made.

## Discussion and conclusions

Paraprostatic cysts are uncommon, and are usually diagnosed in older, large-breed dogs [4, 5]. The prevalence of prostatic cysts in adult large-breed dogs has been reported to be approximately 14%, of which 42% had evidence of bacterial infection [7]. The aetiology of paraprostatic cysts is poorly understood, unlike that of prostatic retention cysts, which usually form as a result of benign hyperplasia of the prostate [13]. Paraprostatic cysts are found outside the prostate and have been found in association with remnants of the *uterus masculinus* [4–10].

The *uterus masculinus* is an embryological remnant of the *paramesonephros* or Müllerian duct system present in males [9]. Its presence is usually asymptomatic, but can cause, on occasions, clinical signs such as urinary incontinence or tenesmus leading to stranguria [14, 15]. *Uterus masculinus* has been well documented in people [16–20], but to our knowledge, only limited reports exist in veterinary medicine [21–23]. In some of the human studies, “*uterus masculinus*” is considered to be the same as enlarged prostatic utricle [16, 18]. In humans, the *uterus masculinus* is described as a hollow structure arising from the craniodorsal aspect of the prostate, from within the genital fold, opening into the prostatic urethra on the *colliculus seminalis* [18]. However, although communication of the cavity of the *uterus masculinus* with the urethra has been reported in horses [24], this has not been reported in dogs. Instead, it has been reported to be attached to the prostate via a stalk of tissue or adhesions [25]. Affected animals may be asymptomatic, but the most commonly reported clinical signs include abdominal distension and/or pain, dysuria and/or haematuria [1, 3–6].

To the authors’ knowledge this is the first documented case of a paraprostatic cyst originating from the *uterus masculinus* that was bacterially infected and communicated with the urethral lumen. The dog presented with clinical signs typical of a paraprostatic cyst: tenesmus and penile discharge. However, an unusual aspect of the presentation was the purulent penile discharge that originated from the infected *uterus masculinus* in the absence of benign prostatic hypertrophy, prostatitis and/or an infection of the urinary bladder.

In addition, this case presented with bilateral Sertoli cell tumour. Similar to other reported cases [11, 21, 22], this case of *uterus masculinus* occurred in an older dog (10 years of age). However, the previous studies mainly reported *uterus masculinus* as an incidental finding during abdominal ultrasonographic examination for other problems [21–23, 26]. The lack of similar clinical signs, such as tenesmus and penile discharge, in the other reported cases may be explained by the presence of cystic *uterus masculinus* in a vestigial state, i.e., without a completely developed lumen or communication with the urethra.

The ultrasonographic appearance of the *uterus masculinus* in the present case was similar to that described in the previous studies [21, 26], with the exception of the presence of hypoechoic, instead of anechoic, fluid in the present case. One previous publication describing *uterus masculinus* hydrometra in a miniature schnauzer and six other dogs reported similar ultrasonographic findings [21, 23].

Histopathology of the *uterus masculinus* in the present case showed the presence of simple columnar epithelium and glandular structures, which is typical of the epithelial lining of the female uterus. Previous reports of the histopathology of *uteri masculini* have described various types of epithelial linings, including pseudostratified columnar, cuboidal and squamous epithelium. This variability is likely to be related to the fact that each *uterus masculinus* developed under different circumstances. The present case also had high circulating concentrations of oestrogen and progesterone, which likely had direct influences on the development of the *uterus masculinus*. High circulating oestrogen concentrations typically accompany testicular Sertoli cell tumours [23, 27]. The source of the progesterone in the present case remains unclear, but the high concentration may also explain the formation of glandular structures within the epithelium and the production of mucus, which is typical of mucometra in female dogs [28]. Finally, the fact that the *uterus masculinus* communicated with the urethral lumen and had a comparable bacterial flora to that of pyometra likely explains the development of a bacterial infection in the present case.

The preferred surgical therapy for a paraprostatic cyst includes exploratory laparotomy with cyst drainage, debulking and either omentalisation or marsupialisation [10, 12, 25]. In the present case, we opted for complete resection of the *uterus masculinus*, following ligation to close its connection with the prostate and urethra, given its infected content.

The case report described here has similarities with the persistent müllerian duct syndrome which is a rare from of pseudohermaphroditism of dogs which is is characterized by XY chromosomal constitution, testes, and feminization of the internal or external genitalia feminized. Commonly these individuals are cryptorchids [23]. However, dog of our case report did not show any signs of feminization or cryptorchidism.

In conclusion, the findings presented in this report suggest the importance of considering a *uterus masculinus* as a differential diagnosis for tenesmus and penile discharge and for structures resembling a paraprostatic cyst. Furthermore, we propose the unification of the terminology regarding *uterus masculinus*, such that the term “true” *uterus masculinus* should be used only if communication with the urethra is present, and “cystic” *uterus masculinus* should be used for cases in which there is no communication with the urethra and columnar glandular epithelium is present. In our opinion, the term “cyst” should be reserved only for an enclosed hollow cavity lined with epithelium and containing a liquid or semi-solid substance [29], and should not be used for structures communicating with the urinary tract, as suggested previously [11, 21–23]. All other structures should be considered to be paraprostatic cysts.

## Data Availability

All data generated or analysed during this study are included in this published article.
